# Analysis of Hendra Virus Fusion Protein N-Terminal Transmembrane Residues

**DOI:** 10.3390/v13122353

**Published:** 2021-11-24

**Authors:** Chelsea T. Barrett, Hadley E. Neal, Kearstin Edmonds, J. Lizbeth Reyes Zamora, Carole L. Moncman, Andreea Popa, Everett Clinton Smith, Stacy R. Webb, Rebecca Ellis Dutch

**Affiliations:** Department of Molecular and Cellular Biochemistry, University of Kentucky, Lexington, KY 40502, USA; ctbarrett05@gmail.com (C.T.B.); hadley.neal@uky.edu (H.E.N.); kearstin.edmonds@uky.edu (K.E.); jlzamora-reyes@uky.edu (J.L.R.Z.); cmonc2@uky.edu (C.L.M.); andreea@aya.yale.edu (A.P.); ecsmith@sewanee.edu (E.C.S.); srwebb4@gmail.com (S.R.W.)

**Keywords:** Hendra virus, fusion protein, transmembrane domain, membrane fusion

## Abstract

Hendra virus (HeV) is a zoonotic enveloped member of the family Paramyoxviridae. To successfully infect a host cell, HeV utilizes two surface glycoproteins: the attachment (G) protein to bind, and the trimeric fusion (F) protein to merge the viral envelope with the membrane of the host cell. The transmembrane (TM) region of HeV F has been shown to have roles in F protein stability and the overall trimeric association of F. Previously, alanine scanning mutagenesis has been performed on the C-terminal end of the protein, revealing the importance of β-branched residues in this region. Additionally, residues S490 and Y498 have been demonstrated to be important for F protein endocytosis, needed for the proteolytic processing of F required for fusion. To complete the analysis of the HeV F TM, we performed alanine scanning mutagenesis to explore the residues in the N-terminus of this region (residues 487–506). In addition to confirming the critical roles for S490 and Y498, we demonstrate that mutations at residues M491 and L492 alter F protein function, suggesting a role for these residues in the fusion process.

## 1. Introduction

Hendra virus (HeV) is a zoonotic, enveloped virus within the viral family Paramyxoviridae. HeV, similar to all enveloped viruses, uses surface glycoproteins to promote binding to a host cell and fusion with host cell membranes to facilitate viral entry [1,2,3,4,5]. More specifically, the HeV attachment protein (G) is utilized for binding the host cell receptor Ephrin B2, while the trimeric transmembrane fusion protein (F) is responsible for merging the viral and host cell membranes [6]. Following proteolytic cleavage, receptor binding, and receipt of a triggering signal, HeV F undergoes a dynamic conformational change enabling the viral and host cell membranes to merge [2,3,4,6]. In order to be cleaved by cellular cathepsins, HeV, and the closely related Nipah virus, F proteins undergo a unique trafficking pattern. They are initially synthesized and trafficked through the secretory pathway to the cellular surface, then are endocytosed to be cleaved by cathepsin L in recycling endosomes before being trafficked back to the cell surface [7,8,9,10,11]. Throughout this trafficking, the F protein remains associated as a trimer [4,5,12,13]. Interactions within the transmembrane (TM) regions of F trimers have been implicated in protein stability and fusion function [11,12,14,15,16]. Additionally, residues S490 and Y498 have been demonstrated to be involved in the endocytosis of F [11]. Previously, double-alanine scanning mutagenesis of the C-terminal end of the HeV F TM region has been performed [15]. While studies have demonstrated several residues of importance in the N-terminal end of the TM [11,12,16], a full characterization of this region has never been performed.

To complement the previous HeV F C-terminal TM domain double alanine-mutagenesis studies [15] and generate a comprehensive characterization of the HeV TM domain, we performed double-alanine scanning mutagenesis of the first 20 amino acids of the TM region (residues 487–506) of HeV F and assessed the effect on protein stability, expression, proteolytic processing, and fusion. Mutants altering residues I489/S490 and L497/Y498 exhibited defects in protein cleavage, surface expression, and fusion. These results are consistent with previous work, showing residues S490 and Y498 play an important role in protein endocytosis [11]. Additionally, a mutant altering S493/M494 demonstrated lower protein surface expression, slightly lower cleavage, and significantly reduced fusion, again consistent with previous work [12]. Interestingly, we found that the alanine mutation of residues M491 and L492 significantly reduced fusion without altering protein expression, newly implicating these residues in the fusion process. We also demonstrate that alanine mutation of the remaining residues does not have an effect on protein expression, stability, or fusion function. The work presented here, when considered with previously published work [7,11,12,15,16], provides a comprehensive analysis of the entire HeV F TM region and identifies the critical residues within it.

## 2. Materials and Methods

### 2.1. Cell Lines and Culture

Vero cells (ATCC) and BSR T7/5 cell lines (obtained from Dr. Karl-Klaus Conzelman, Pettenkofer Institut) were maintained in Dulbecco’s modified Eagle’s medium (DMEM; GE Healthcare) supplemented with 10% fetal bovine serum (FBS; Sigma, Burlington, MA, USA). For the BSR T7/5 cell line, 0.5 mg/mL G-418 sulfate (Invitrogen, Waltham, MA, USA) was added to the medium every third passage to select for T7 polymerase expressing cells. 

### 2.2. Plasmids and Antibodies

The genes for Hendra F or G, derived from plasmids provided by Dr. Lin-Fa Wang (Australian Animal Health Laboratory), were subcloned into pCAGGS either as described in [10] or using the forward primer 5′-GCG ATT GAA TTC TAA GCA ATG GCT ACA CAA GAG-3′ and reverse primer 5′-CG GCG GCC ATG CAT ATT TTA TGT TCC AAT ATA ATA-3′ for PCR amplification [14]. All Hendra F mutants were made in pGEM using the QuikChange site-directed mutagenesis kit (Stratagene, La Jolla, CA, USA), and subcloned into the eukaryotic expression vector pCAGGS [17]. Antibodies used included a polyclonal antibody (Genemed Custom Peptide Antibody Service, San Francisco, CA, USA) generated to residues 516–529 of Hendra F and a mouse monoclonal antibody to Hendra F termed 7F7 (kindly provided by Dr. Christopher Broder, Uniformed Services University). 

### 2.3. Syncytia Assay

Vero cells (70–90% confluent) in 6-well plates were transiently transfected with pCAGGS-Hendra F in combination with the attachment protein, pCAGGS-Hendra G. Transfections were performed at a ratio of 1:3 (F:G) using Lipofectamine 3000 system (Invitrogen) according to the manufacturer’s protocol. Syncytia formation was examined at 24 and 48 h post transfection (hpt) and images were taken using a Nikon digital camera mounted atop a Nikon Ti2 microscope with a 20× objective. 

### 2.4. Luciferase Reporter Gene Assay

Vero cells (70–90% confluent) in 12-well plates were transiently transfected with 0.15 μg of pCAGGS-Hendra F or one of the HeV F mutants, 0.45 μg of pCAGGS-Hendra G, and 0.4 μg of a T7 promoted luciferase plasmid. Transfections were performed using Lipofectamine and Plus reagent (Invitrogen) according to the manufacturer’s protocol. At 24 hpt, BSR T7/5cells were lifted with trypsin, resuspended in DMEM supplemented with 10% FBS, and overlaid onto Vero cells that were washed once with PBS. Cells were incubated for 4 h at 37 °C, then were lysed with reporter lysis buffer (Promega, Madison, WI, USA) and analyzed for Luciferase activity using the luciferase assay system (Promega) per manufacturer’s instructions. A SpectraMax iD3 plate reader (Molecular Devices; Sunnyvale, CA, USA) was used to quantify the luminescence readout. Results were normalized to cells expressing WT Hendra F and G, after subtracting the background (HeV G only). 

### 2.5. Surface Biotinylation

Vero cells were plated in 6-well plates and transiently transfected using Lipofectamine 3000 reagent, per the manufacturer’s protocol, with 2 μg of WT pCAGGS-HeV F or one of the TM mutants. At 24 hpt, cells were washed with PBS and starved for 45 min in DMEM deficient in cysteine and methionine (Cys^−^/Met^−^ DMEM). The samples were radiolabeled for 3 h with Cys^−^/Met^−^ DMEM containing Tran-^35^S (100 μCi/mL; PerkinElmer, Waltham, MA, USA). The cells were then washed twice with 1 mL ice cold PBS (pH 8.0) and biotinylated for 35 min at 4 °C with rocking using 1 mL of 1 mg/mL EZ-Link-Sulfo-NHS-Biotin (Thermo Scientific, Waltham, MA, USA) in PBS (pH 8.0). This was followed by an incubation at room temperature for 15 min. Cells were washed twice with ice cold PBS (pH 8.0) and lysed with 500 ul RIPA buffer (100 mM Tris-HCl [pH 7.4], 0.1% SDS, 1% Triton X-100, 1% deoxycholic acid) containing 150 mM NaCl, protease inhibitors (1 U aprotinin, 1 mM PMSF, [both from Sigma-Aldrich, Burlington, MA, USA]), 5 mM iodoacetamide (Sigma-Aldrich), and cOmplete EDTA-free Protease Inhibitor Cocktail Tablets (Sigma-Aldrich). Cell lysates were centrifuged at 136,500× *g* for 15 min at 4 °C. The supernatant was transferred to 1.5 mL tubes and incubated with 4 µL of Hendra F antibody for 3 h at 4 °C with rocking. Proteins were immunoprecipitated by incubating each sample with 30 µL protein A-Sepharose beads (GE Healthcare, Chicago, IL, USA) for 30 min at 4 °C with rocking. The beads were washed twice with RIPA buffer plus 0.30 M NaCl, twice with RIPA buffer plus 0.15 M NaCl, and once with SDS wash II (150 mM NaCl, 50 mM Tris-HCl, pH 7.4, 2.5 mM EDTA). Following the final wash, 60 µL of 10% SDS was added and the samples were boiled for 10 min, spun down, and then the supernatant was transferred to a new tube, and repeated with 40 µL of 10% SDS for a total of 100 µL. Ten microliters of each sample was separated and added to 10 μL 2× sample loading buffer and labeled “TOTAL”. The remaining sample was labeled “SURFACE” and incubated with 400 µL biotinylation dilution buffer (20 mM Tris [pH 8.0], 150 mM NaCl, 5 mM EDTA, 1% Triton X-100, 0.2% bovine serum albumin (US Biological Life Sciences, Salem, OR, USA)) and 30 µL streptavidin beads (Thermo Scientific) for 1 h at 4 °C with rocking. The washes described previously were repeated and 30 μL of 2× sample loading buffer was added following the final wash. Samples were boiled, run on a 10% SDS-PAGE, dried, exposed on a phosphor screen, and visualized using the Typhoon imaging system (GE Healthcare). Band densitometry using ImageQuant version 5.2 was performed for each experiment to quantitate the relative percent expression of F compared to WT HeV F. The quantification is the sum of F_0_ and F_1_ for each sample and the percent cleavage is calculated by dividing F_1_ by the sum of F_0_ and F_1_.

### 2.6. Time Course Immunoprecipitation

Vero cells were plated in 6-well plates and transiently transfected using Lipofectamine 3000 reagent per the manufacturer’s protocol with 2 μg of WT pCAGGS-HeV F or one of the TM mutants. At 24 hpt, cells were washed twice with PBS and starved with Cys^−^/Met^−^ DMEM for 45 min at 37 °C. Cells were then metabolically labeled with Cys^−^/Met^−^ DMEM containing Trans-^35^S (50 µCi/mL; PerkinElmer) for 1 h. Following the label, cells were washed twice with PBS, then chased with DMEM (10% FBS, 1% penicillin-streptomycin) for 1, 2, 4, 8, and 24 h. At the appropriate timepoints, chase media was aspirated and cells were washed three times with PBS. The cells were lysed with RIPA buffer and frozen at −20 °C. The samples for the 0 h timepoint were immediately lysed with 500 µL of RIPA lysis buffer and frozen after the label period. Cell lysate was immunoprecipitated with the Hendra F polycloncal antibody and sepharose-A beads and protein was visualized as described for surface biotinylation.

### 2.7. High Molecular Weight Immunoprecipitation and Non-Reducing Gel Electrophoresis

Hendra F or the TM mutants were expressed in Vero cells using the Lipofectamine 3000 system (Invitrogen), as previously described. The next day cells were washed with PBS and starved for 45 min at 37 °C in Cys^−^/Met^−^ DMEM. Cells were then labeled with Trans-^35^S in Cys^−^/Met^−^ DMEM (50 μCi/mL) for 4 h, washed two times with PBS, and lysed with RIPA lysis buffer. Cell lysates were incubated with the Hendra F polyclonal antibody for 3 h, then with protein A-Sepharose Beads for 30 min, all at 4 °C. The same washes were performed as described for surface biotinylation. After washing steps were complete and the final wash aspirated, 30 μL of 2× SDS loading buffer without dithiothreitol (DTT) was added to each sample. Samples were then incubated at 60 °C or 100 °C, as indicated, for 15 min and run on a 3.5% acrylamide gel under non-reducing conditions. The gel was imaged using the same system described for surface biotinylation.

### 2.8. Immunofluorescence

Vero cells seeded on coverslips were transiently transfected using Lipofectamine 3000 reagent per the manufacturer’s protocol with 2 μg of WT pCAGGS-HeV F, or one of the TM mutants. At 24 hpt, samples were washed twice with 1 mL of PBS plus 0.02% sodium azide (PBSN) and fixed in 4% paraformaldehyde in PBSN at room temperature for 15 min. Samples were then washed three times with 1 mL of PBSN and permeabilized with 1 mL of PBSN and 1% Triton X-100 for 15 min at 4 °C. Samples were again washed three times with PBSN and then moved from the wells into a humidified chamber. Coverslips were blocked with 1% normal goat serum (NGS) in PBSN for 1 h at 4 °C. Blocking buffer was removed and primary antibody (7F7; 1:1000 dilution) in 1% NGS in PBSN was added. Samples were incubated overnight at 4 °C. Following primary antibody label, coverslips were washed 7X with PBSN plus 0.05% Tween, then incubated with goat anti-mouse FITC (1:1000 dilution) in 1% NGS in PBSN for 1 h at 4 °C. Coverslips were washed 7X with PBSN plus 1% NGS and mounted on glass slides using 1.5 μL of VECTASHIELD mounting media (Vector Labs, San Francisco, CA, USA). Images were taken using an Axiovert 200 M microscope (Zeiss, Oberkochen, Germany) at 40× magnification, using Metamorph to capture z-stacks, and processed with NIS Elements.

## 3. Results

**Figure 1:**
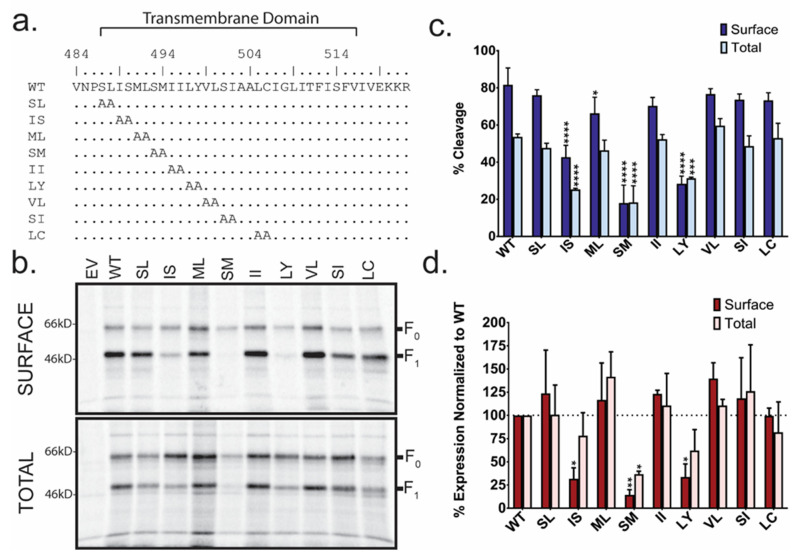
Several Hendra virus (HeV) F transmembrane (TM) mutants have impaired protein expression and protein cleavage. (**a**) Alanine scanning mutations at the N-terminus of the HeV F TM were generated to assess the importance of these residues. The putative boundaries of the TM are indicated. (**b**) Surface biotinylation was performed to analyze the surface and total population of HeV F for empty vector (EV), WT and each of the mutants created. Cells were radiolabeled for 3 h. Using band densitometry, percent expression (**c**) and percent cleavage (**d**) was measured for the total and surface protein. All measurements represent the average of three independent experiments ± SD, and all samples were normalized to WT. Significance was determined by two-way analysis of variance (ANOVA) * = *p* < 0.05, ** = *p* < 0.01, *** = *p* < 0.005, **** = *p* < 0.001.

**Figure 2:**
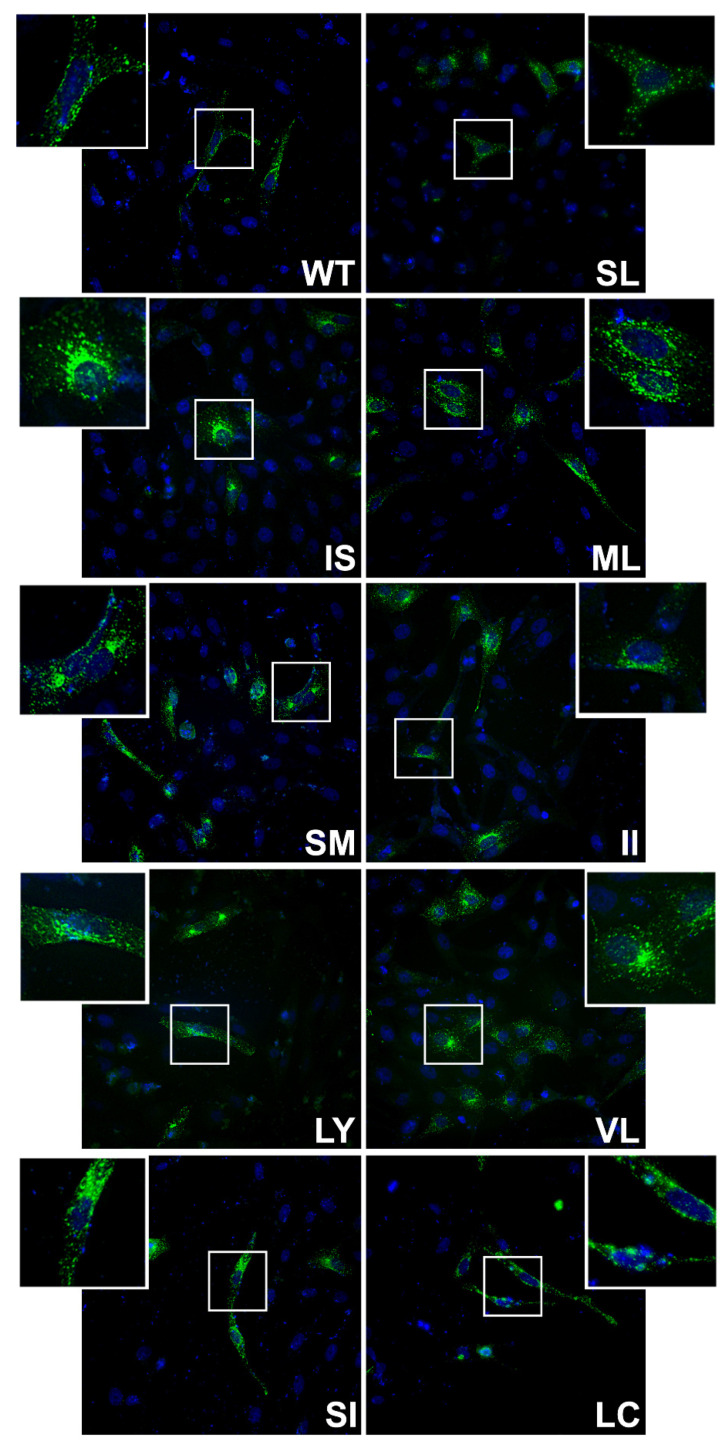
HeV F TM mutants have a similar distribution to WT F in immunofluorescence. Immunofluorescence of WT HeV F or each of the mutants created (Green is F, Blue is DAPI) transiently expressed in Vero cells. Insets represent enlargements of the boxed areas.

To assess the potential role of residues at the N-terminus of the HeV F protein TM region, double alanine scanning mutants were created (Figure 1a). Notably, there is a naturally occurring double alanine (residues 503–504) within the TM region to which changes were not made. Surface biotinylation was then performed to determine the surface and total protein population expression, and cleavage levels of transiently transfected wild-type (WT) HeV F or mutant F (Figure 1b). WT HeV F and most TM mutants were detected after immunoprecipitation as two bands, corresponding to F_0_ (un-cleaved F) and F_1_ (cleaved F) (Figure 1b). Using band densitometry, percent expression (Figure 1d) and percent cleavage (Figure 1c) were calculated for both surface and total protein populations and normalized to WT HeV F. Mutants IS, SM, and LY all showed reduced surface expression compared to WT F (Figure 1d; *p* < 0.05, *p* < 0.01, *p* < 0.05 respectively). SM also demonstrated lower total protein expression (Figure 1d; *p* < 0.05). Additionally, mutants IS, SM, and LY all had a significant reduction in protein cleavage in both surface and protein populations (Figure 1c). Interestingly, the ML mutant had a slight, but significant, reduction in cleavage in the surface protein population (Figure 1c; *p* < 0.05), while showing no cleavage differences in the total protein or overall protein expression (Figure 1d). This suggests, consistent with previous studies, that mutants IS, SM, and LY have defects in the endocytosis process needed for proteolytic processing. When WT or mutant F protein expression was assessed using immunofluorescence, the WT F protein showed a pattern of staining consistent with known trafficking patterns through the secretory pathway, cell surface, and endocytic pathway. Mutants IS, SM, LY, SI, and VL demonstrated more staining near the nucleus (IS and SM demonstrate a robust localization near the nucleus), although some staining was observed throughout the cell in all samples (Figure 2), indicating an alteration in protein trafficking and/or localization due to these mutations.

**Figure 3:**
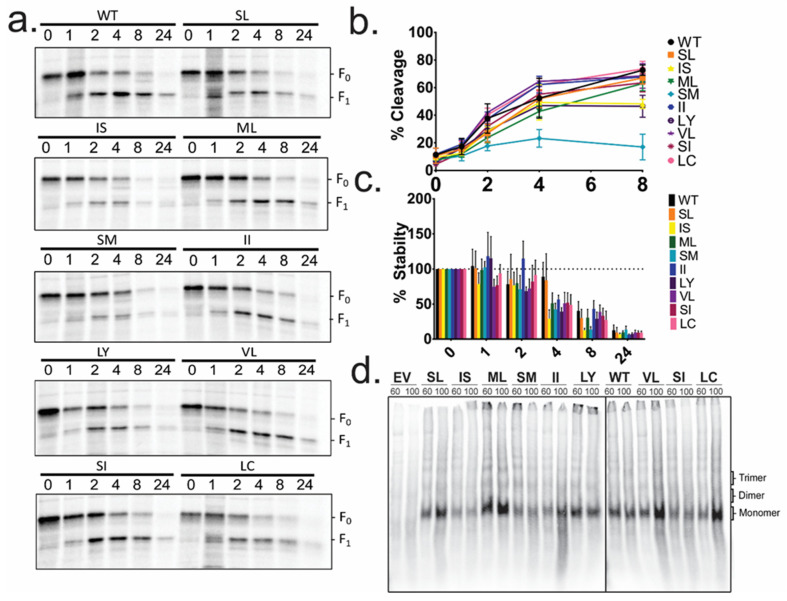
HeV F TM mutations at residues SM and LY decrease protein cleavage levels. (**a**) Vero cells were transfected with WT HeV F or the TM mutant DNA, metabolically labeled for one hour, and chased for times indicated (hours). Using band densitometry (**b**) percent cleavage and (**c**) percent stability were calculated. Graphs are shown as the average of three independent experiments ± SEM. (**d**) Vero cells transiently expressing empty vector (EV), WT F (WT), or each TM mutant were metabolically labeled for 6 h. Samples were treated at the indicated temperatures before separation on a non-reducing SDS-PAGE. Oligomers are labeled on the right based on size (*n* = 3). Two gels were run under the same conditions to accommodate all samples. A black line is used to separate the lanes that were run on the second gel.

Pulse-chase analysis was utilized to examine WT F or mutant protein cleavage kinetics and protein stability (Figure 3a). Most TM mutants displayed no change in protein cleavage compared to WT F. Mutants SM and LY however, showed decreased cleavage at later time points. Both mutants also demonstrated decreased protein cleavage and protein expression in the surface biotinylation experiment (Figure 1c,d). The SM mutant demonstrated a significant reduction in cleavage compared to WT F at four and eight hours of chase (*p* < 0.01, *p* < 0.0001, respectively). The LY mutant exhibited a reduction in cleavage at 8 h of chase (*p* < 0.01). However, all TM mutants had similar protein turnover kinetics compared to WT F, with protein remaining stably expressed through 2 h of chase. After 4 and 8 h of chase, only about 40–50% of protein remained, and only about 10–15% remained after 24 h (Figure 3c). This suggests that even mutations that alter F protein cleavage efficiency did not significantly disrupt protein stability.

HeV F associates as a trimer shortly after synthesis and remains in a trimeric association throughout protein trafficking and fusion. HeV F TM–TM interactions have been shown to be important in this trimeric association and stability [11,12,15,16]. Therefore, to assess the effect the TM mutations had on the stability of oligomers of WT HeV F, or each of the mutants, was transiently expressed and metabolically labeled. Following cell lysis, and immunoprecipitation, samples were treated at 60 °C or 100 °C prior to separation on non-reducing SDS-PAGE. After either temperature treatment, WT HeV F migrated primarily as a monomer, although some dimer and trimer species were observed (Figure 3d). Similar migration patterns were observed for each of the TM mutants, suggesting that the introduced mutations were still able to form oligomeric species of the protein.

**Figure 4:**
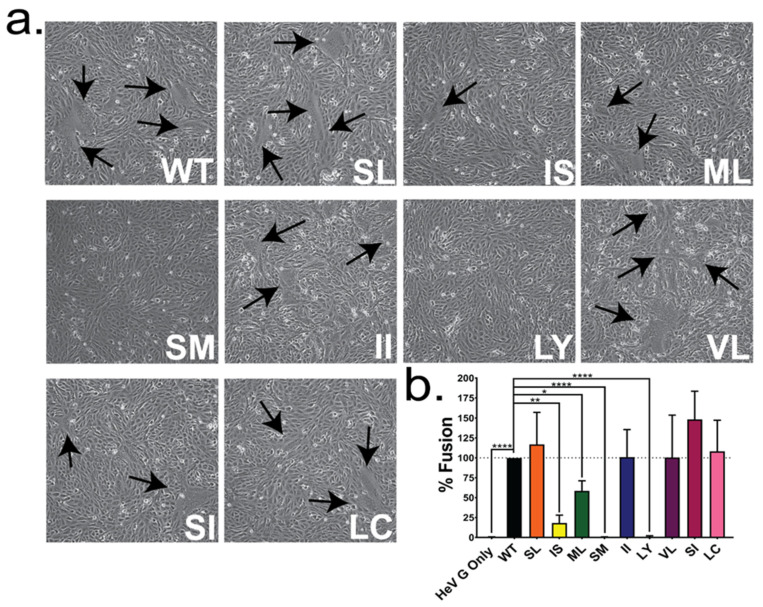
Mutations at residues IS, ML, SM, and LY in the HeV F TM reduce fusion. (**a**) Vero cells were transfected with the HeV attachment protein (G), and WT HeV F or one of the TM mutants. Syncytia formation was analyzed at 48 hpt. Black arrows indicate syncytia formation. Images are representative of 4 independent experiments. (**b**) A luciferase reporter gene assay was performed using BSR/T7 cells overlaid onto Vero cells transfected with HeV G and WT HeV F or each of the TM mutants at 24 hpt. Results are normalized to samples with WT HeV F and G and representative of 3 independent experiments, performed in duplicate. Significance was determined by one-way ANOVA. * = *p* < 0.05, ** = *p* < 0.01, **** = *p* < 0.001.

After characterizing the expression and proteolytic processing of the HeV F TM mutants, we then examined the overall effects on fusion function of HeV F using syncytia formation assays and luciferase reporter gene fusion assays. For the syncytia assay, WT HeV F or the TM mutants were transiently co-expressed with HeV G and imaged at 48 h (Figure 4a). The formation of large syncytia was observed in WT samples, as well as mutants SL, II, VL, SI, and LC. Very few syncytia were observed in samples IS and ML, and no syncytia were found in samples with mutant SM or LY (Figure 4a). To quantitate the levels of fusion, a luciferase reporter gene assay was employed by transiently co-expressing WT HeV F or each TM mutant with HeV G and luciferase under the control of a T7 promoter in Vero cells, and overlaying them with BSR/T7 cells. All samples were normalized to samples expressing WT HeV F and G. Similar to the syncytia results, the HeV F mutants SL, II, VL, SI, and LC demonstrated fusion levels similar to WT (Figure 4b). In contrast, the mutants HeV F SM and LY did not exhibit any fusion above background levels (*p* < 0.0001), and mutants IS and ML exhibited significantly reduced fusion levels (20% [*p* < 0.01] and 50% [*p* < 0.05], respectively) compared to WT. The reduction in fusion observed for mutants IS, SM, and LY is consistent with the significantly reduced protein expression and cleavage observed for these mutations (Figure 1c,d). However, the slight reduction in cleavage (10%) observed for the surface population of ML is not enough to account for the 50% reduction in fusion, since previous work has shown that the amount of cleaved protein at the cell surface directly correlates to the amount of fusion observed [18].

## 4. Discussion

Multiple studies have indicated that the TM domain of class I fusion proteins plays an important role in proper protein folding and function [1,4]. The F proteins of many enveloped viruses have specific sequence requirements for their TM domain including SARS Coronavirus, human Immunodeficiency Virus-type 1, parainfluenza virus 5, and Newcastle disease virus. [19,20,21,22,23]. Alterations to the TM sequence in these viruses impacts fusion activity. Specifically, substituting the TM domain of the SARS Coronavirus (SARS-CoV) spike protein (S) aromatic region with alanine residues abolished membrane fusion without effecting overall cell surface expression [20]. Similarly, the mutation of the membrane spanning domain (MSD) within the Human Immunodeficiency Virus-type 1 (HIV-1) envelope glycoprotein to form a leucine-rich hydrophobic core resulted in defective infectivity [23].

This sequence dependency could be localized to specific regions of the TM domain. One study in parainfluenza virus 5 (PIV5) concluded that the F protein TM domain acts in a sequence dependent manner with β-branched residues at the N-terminal region [19]. Using alanine scanning mutagenesis, it was determined that mutating N-terminal leucine and isoleucine residues resulted in a significant decrease in fusion activity [19]. Additionally, analysis of the C-terminal residues of the HeV F TM domain indicated that when β-branched residues were mutated to alanine, the protein became hypofusogenic [15], suggesting sequence dependency localized to the C-terminal HeV F TM domain [15]. However, the data presented in this manuscript reveals a lack of sequence dependency for β-branched residues on the N-terminus of the HeV F TM.

The role of several residues within the N-terminus of the TM of HeV F have been previously explored. Residues L488 (within mutant SL), I495 (within mutant II), and I502 (within mutant SI) were determined to be part of a Leucine-Isoleucine zipper (LI Zipper) motif that runs through the TM region [16]. Single mutations of each of these residues resulted in only minor protein expression, cleavage, and fusion activity changes [16], consistent with our study. However, mutation of the entire LI zipper resulted in a significantly destabilized pre-fusion conformation, thus abolishing the fusion activity of the protein [16]. Interestingly, including mutations of the neighboring residues (S487, I496, S501) with mutations to the LI Zipper had no appreciable effect on protein expression, stability, or fusion function suggesting that these serine residues and isoleucine residue are dispensable for HeV F function (Figure 1, Figure 3 and Figure 4). Residues S490 (within mutant IS) and Y498 (within mutant LY) were demonstrated to play a role in protein endocytosis and subsequent recycling, with the hydroxyl group and aromatic nature being critical in each residue, respectively [11]. Efficient endocytosis and recycling of the HeV F protein was also shown to be critical for proper virus-like particle assembly, as endosomes are the likely location of HeV F association with HeV matrix protein during particle assembly [7]. Interestingly, inclusion of the neighboring residues methionine and leucine allowed for a modest increase in protein cleavage and protein stability compared to previous work on the single point mutations [11]. This suggests that mutation of the two neighboring residues may have a partial compensatory effect, potentially restoring some of the protein endocytic recycling. Residue S493 (within mutant SM) has been demonstrated to have reduced cell surface expression and fusion levels by about 80%, in a previous study [12]. Interestingly, when we included the neighboring methionine residue in the mutation, cell surface expression was further reduced and fusion activity was abolished, indicating a compounding effect when both the S and M are mutated. This may indicate both of these residues play critical roles in fusion, protein conformation, and stability.

Residues M491 and L492 (mutant ML) had not previously been explored in the context of HeV F. Residue M491 is predicted to lie on the contact interface between the protomers in the trimeric protein [11], suggesting it may have a role in TM–TM associations of this protein. While the M491A/L492A mutant is still able to form protein oligomers (Figure 3d), it does exhibit a significant reduction in protein fusion (Figure 4b). The mutation of the large methionine and β-branched leucine residues to alanine residues may cause the protein trimer to pack together too tightly disrupting the TM-TM dissociation needed to carry out fusion [14]. Alternatively, this mutation could increase the flexibility of the protein in this region, loosening TM-TM associations and causing the TM regions to dissociate too much during the fusion process. This dissociation would destabilize protomer association during intermediate steps of fusion. There is also potential that these mutations disrupt interactions with the fusion peptides that are needed for fusion pore expansion.

In addition to mutation of residues M491 and L492, this work also represents the first-time residues V499/L500 and L505/C506 were investigated. When the C-terminal end of the HeV F TM region was studied, mutations of β-branched residues appeared to play a critical role. Interestingly, both mutant VL and LC, which lie in the middle of the predicted HeV F TM and contain β-branched residues, demonstrate no change to protein expression or fusion activity, indicating these residues are not critical. This suggests that these β-branched residues play a larger role in the C-terminus of the HeV F TM, potentially contributing to an increase in flexibility needed by that end [15]. This work, when partnered with previous studies [7,11,12,15,16], completes characterization of the entire HeV F TM region. While this work has investigated HeV F protein stability, expression, and cell–cell fusion function, understanding the interactions of HeV F TM residues with viral proteins other than HeV G, or host proteins and membrane lipids remains to be explored.

## Data Availability

Data is available upon request from the corresponding author.
